# An accurate coarse-grained model for chitosan polysaccharides in aqueous solution

**DOI:** 10.1371/journal.pone.0180938

**Published:** 2017-07-21

**Authors:** Levan Tsereteli, Andrea Grafmüller

**Affiliations:** Theory and Bio-Systems, Max Planck Institute of Colloids and Interfaces, Potsdam, Germany; University of Lincoln, UNITED KINGDOM

## Abstract

Computational models can provide detailed information about molecular conformations and interactions in solution, which is currently inaccessible by other means in many cases. Here we describe an efficient and precise coarse-grained model for long polysaccharides in aqueous solution at different physico-chemical conditions such as pH and ionic strength. The Model is carefully constructed based on all-atom simulations of small saccharides and metadynamics sampling of the dihedral angles in the glycosidic links, which represent the most flexible degrees of freedom of the polysaccharides. The model is validated against experimental data for Chitosan molecules in solution with various degree of deacetylation, and is shown to closely reproduce the available experimental data. For long polymers, subtle differences of the free energy maps of the glycosidic links are found to significantly affect the measurable polymer properties. Therefore, for titratable monomers the free energy maps of the corresponding links are updated according to the current charge of the monomers. We then characterize the microscopic and mesoscopic structural properties of large chitosan polysaccharides in solution for a wide range of solvent pH and ionic strength, and investigate the effect of polymer length and degree and pattern of deacetylation on the polymer properties.

## Introduction

Chitin is one of the most abundant natural biopolymers and the most abundant amino-polysaccharide on the planet [1]. Its main derivative, Chitosan, is produced by N-deactylation of the 2-acetamide-2-deoxy-*β*-d-glucopyranose (GlcNac) monomers. Thus Chitosan is composed of (1-4) linked units of 2-amino-2-deoxy-*β*-d-glycopyranose (GlcN) as well as, because deacetylation is never complete, GlcNac monomers. Typically, the polymers are referred to as chitosan if the degree of deacetylation (DD) is larger than 50%. The amino groups of the deacetylated monomers can be protonated in mildly acidic conditions, making the polymers soluble and Chitosan one of the rare cationic polymers [2]. Due to the weakly ionic nature of the GlcN monomers, which exist either as neutral GlcNH_2_ or positively charged ${\text{GlcNH}}_{3}^{+}$, the DD, as well as the pysico-chemical conditions of the solution strongly influence polymer solubility, size, flexibility and aggregation [3–7].

As it is readily available and possesses many desirable properties including bio-compatibility, bio-degradability and low nanotoxicity, Chitosan has found many applications in food, cosmetic, biomedical and pharmaceutical industry [4, 8, 9]. Particular interest has emerged in using Chitosan in drug delivery systems over the last decade [10].

A precise understanding of the relation between structural characteristics of Chitosan in aqueous solution and these factors is of great interest for the efficient engineering of Chitosan based materials. In crystalline Chitosan, the polymers typically have an extended twofold helical structure [11–13] similar to that of *α*-Chitin, however different types of helical structures are also observed for certain experimental conditions [11–17]. Information on the local structure of the sugars in aqueous solution on the other hand can only be inferred by indirect methods, leading to sometimes contradictory results [18–20].

Molecular simulations can provide valuable insights into the molecular structure under different conditions. However, the large size and complex interactions of polysaccharides present severe limitations for accurate computational modeling. To make matters more complicated, the strong pH dependence of the amino group charge requires careful treatment. Nonetheless, all-atom models have been applied to study the conformational flexibility of different chitosan oligosaccharides [21] and the interactions of infinite filaments [22, 23].

Coarse grained (CG) simulation models, which group a number of atoms together into one interaction center can overcome some of the above limitations, however, at the cost of the detailed chemical information. Different strategies for the parametrization of bottom-up CG models for different polysaccharides, which preserve as much chemical detail of the atomistic system as possible have been pursued in the past [24–27]. In addition, the popular top-down MARTINI CG model [28–30] has recently been extended to include parameters specifically for modeling the *α*-Chitin [31] crystal and for GlcNac and ${\text{GlcNH}}_{3}^{+}$ [32] monomers, which have been used to model chitosan self assembly with discontinuous molecular dynamics simulations.

**Fig 1:**
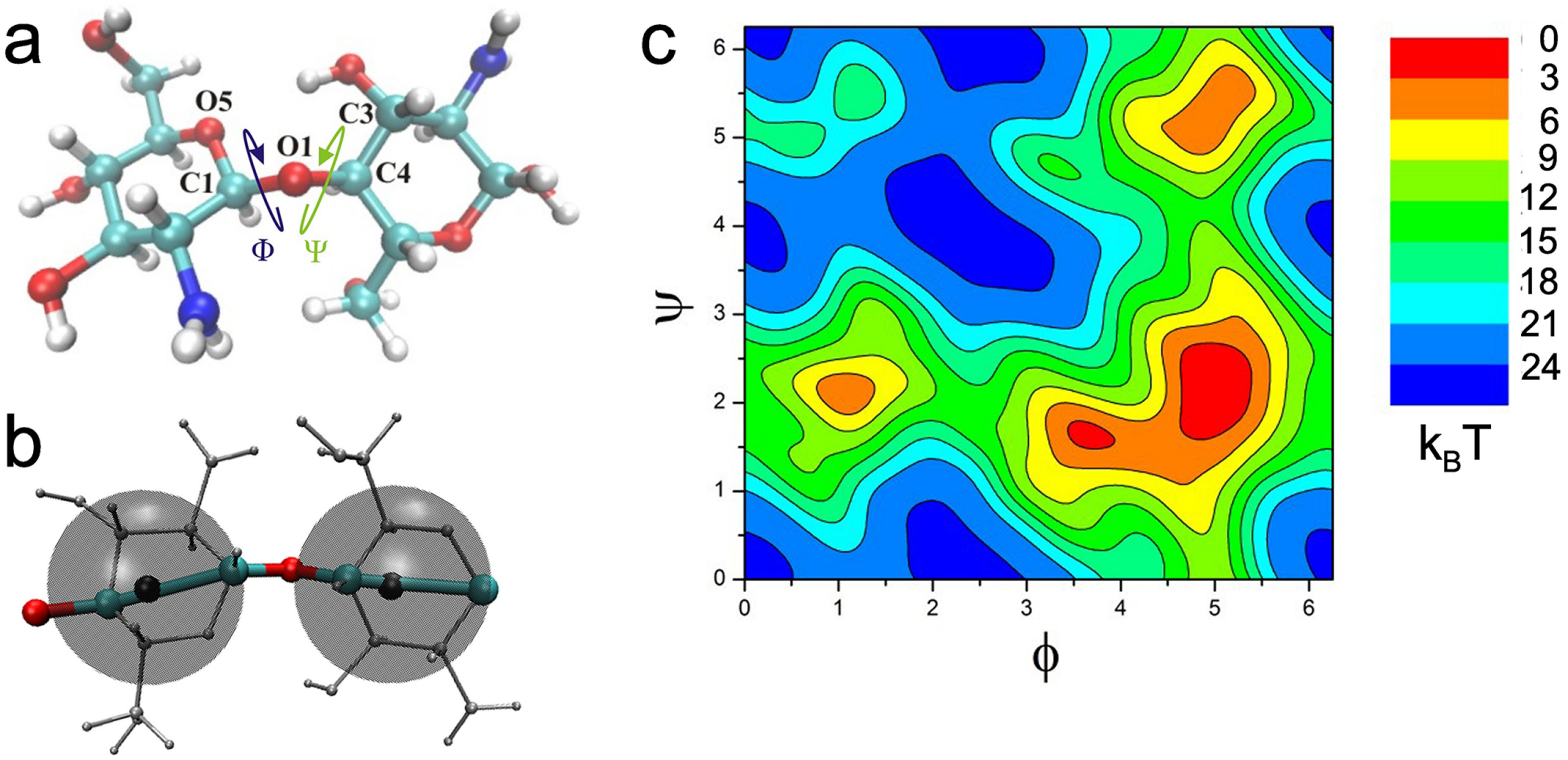
The glycosidic link in a GlcNH_2_ -GlcNH_2_ dimer. a) The dihedral angles *ϕ* and *ψ*. Atoms defining *ϕ* and *ψ* are lablled. b) CG Topology. Atoms and bonds in the CG Model are drawn thick and in color. The all atom structure of the monomers is shown in gray for orientation. The centers of the CG interaction sites are shown in black, while the transparent gray spheres illustrate the range of steric interactions. c) A free energy map for *ϕ* and *ψ*.

One strategy that has been effective for sampling the conformations of large polysaccharides in solution, is based on the conformational preferences of the most flexible degrees of freedom, the glycosidic angles *ϕ* and *ψ* shown in Fig 1 [33–39]. These models make use of the comparatively stiff nature of the bulky sugar rings. Together with the propensity for internal hydrogen bond formation, this leads to a strong conformational preference and interdependence of *ϕ* and *ψ*, which is incorporated in the model via potential of mean force (PMF) maps obtained from all atom sampling methods.

Here, we describe and validate a similar CG model for the precise characterization of Chitosan polysaccharides in solution, which samples the conformations of all glycosidic bonds using pivot move Monte Carlo (MC) simulations. The monomers are mapped into one CG interaction center, on which steric and electrostatic interactions act. PH and ionic strength of the solution enter the model via titration moves on the GlcN monomers, taking into account both the intrinsic pKa value of the monomers and the local electrostatic environment created by nearby charges. We observe that the details of the glycosidic PMFs can significantly affect the structural characteristics of long polymer chains. Furthermore, a change in the charge state of a GlcN Monomer may affect the free energy map of the glycosidic links at both ends, slightly shifting the position of the minima and their relative depths. Therefore, we calculated a complete set of nine different PMF maps depending on the monomer types and charge state at both ends of the glycosidic link. In a protonation move, the corresponding dihedral maps are adjusted according to the new charge state. This procedure is found to greatly improve agreement with experimental observables.

In the following sections, first a detailed description of the model, CG force field, and simulation protocol is given. Then the model is validated against equilibrium properties of chitosan polymers and applied to systematically investigate their dependence on degree of polymerization (DP), degree and pattern of deacetylation, ionic strength and pH.

## Methods

### Molecular dynamics simulations

All-Atom MD simulations were used to define the topology and bonded interactions of the CG model. Simulations were performed in GROMACS 4.6.1 [40, 41], using the GLYCAM06 [42, 43] force field for the sugars and a number of different water models, *i.e.* SPC [44], SPCE [45], Tip3p, Tip4p [46], Tip4pew [47] and Tip5p [48].

The sugar molecules were solvated in a cubic box of 4x4x4 nm. Initial structures were energy minimised by 100000 steps of steepest descent followed by 200 ps equilibration using the stochastic dynamics integrator with a 2 fs timestep at a temperature of 298 K. The pressure was set to 1 bar with the berendsen barostat [49]. Bonds involving hydrogens were constrained with LINKS [50], water molecules were kept rigid using SETTLE [51]. A cut-off of 1.4 nm was used for coulomb and LJ interactions. Long range electrostatics were treated with the Particle Mesh Ewald (PME) method [52].

Metadynamics simulations [53–55] were performed using the PLUMED plug-in version 2.2.1 [56], to calculate the PMF maps of the glycosidic rotational angles *ϕ* and *ψ*. For computational efficiency, Metadynamics on a grid was used with 200 bins for each angle. To obtain smooth sampling, a deposition frequency of 25000 steps, i.e. 50 ps was used with a Gaussian width of 0.25 Rad, which corresponds to half of the standard deviation of the fluctuations of the dihedral angles in the unbiased simulations and a Gaussian potential height of 0.5 kJ/mol. The Maps were sampled for 200 ns.

Previous MD simulations found that each glycosidic link is independent of its neighbors [57], and PMF maps calculated here for DP up to 16 Monomers showed no significant polymer length dependence. Therefore a set of nine PMF maps corresponding to all possible combinations of GlcN and GlcNac were calculated using dimers. The IUPAC definition for the dihedral angles *ψ* and *ϕ* was used [58].

### Coarse-Grained topology

The CG polymer model makes use of the restricted conformational space of *ψ* and *ϕ*. The topology of the CG model retains the atoms defining the two dihedrals, illustrated by the thick bonds in Fig 1(b). All bonds and angles are kept constant, so that the only flexible degrees of freedom are the dihedral angles of the glycosidic link. Polymer conformations are sampled according to the free energy maps for the glycosidic dihedral angles, such as the one shown in Fig 1c for GlcNH_2_ -GlcNH_2_. The atoms of the carbohydrate rings are mapped into additional CG interaction sites, on which steric and electrostatic forces act. These are centered on the average center of geomerty of the ring atoms for the QM optimized structure of the sugar monomer.

### Force field definition

The interactions that describe the CG forcefield are bonded interactions along the polymer and non-bonded interactions between remote polymer sites. Water molecules are not explicitly represented in the model. The effect of hydration are implicitly included via the glycosidic PMF maps. The details of the different interaction potentials are described in the following:

*Bonded interactions* between successive monomers are represented by the free energy maps of the glycosidic torsion angles *ψ* and *ϕ*. These maps capture the strong interdependence between the two angles, and indirectly incorporate all the factors contributing to the conformations of *ψ* and *ϕ* at the atomistic scale, such as van der Waals and electrostatic interactions between the bulky rings, hydrogen bond formation, solvation effects and conformational entropy of the link. The strong conformational restriction of the glycosidic links is reflected in the relatively small areas of the minima.

*Excluded volume* effects were included using a repulsive Lennard-Jones (LJ) potential of the form

$$\begin{array}{c} U^{LJ}=\{\begin{array}{cc} 4\varepsilon^{\text{LJ}}\left[{\left(\frac{\sigma^{\text{LJ}}}{r}\right)}^{12}-{\left(\frac{\sigma^{\text{LJ}}}{r}\right)}^{6}+\frac{1}{4}\right] & r\leq r_{c}\\ 0 & r>r_{c} \end{array} \end{array}$$ (1)

where *r* is the distance between the monosaccharide centers, *ε*^LJ^ and *σ*^LJ^ are the LJ energy parameter and radius, respectively and *r*_*c*_ = 2^(1/6)^ *σ*^*LJ*^ is the cutoff radius, corresponding to the minimum of the LJ potential. *σ*^LJ^ was chosen such that the surface area of the LJ sphere is equivalent to the molecular surface area of the monomer. As the LJ interactions are purely repulsive, results are very insensitive to the value of *ε*^LJ^ and thus the parameters for carbon *ε*^LJ^ = 0.6276 kJ/mol was chosen. Lorentz-Berthelot mixing rules were used.

*Electrostatic interactions* between non-adjacent charged monomers in aqueous solutions of different ionic strengths were modeled with Debye-Huckel interactions.

$$\begin{array}{c} U_{\alpha \beta }^{DH}=z_{\alpha }z_{\beta }k_{B}T\lambda_{B}\frac{e^{-\kappa r}}{r} \end{array}$$ (2)

where *z*, equal to 0 or 1, is the valence of the chitosan monomers, *λ*_*B*_ is the Bjerrum length equal to 7.14 Åin water at 298 K and *κ*^−1^ is the Debye length. The ionic strength *c*_s_ of the solution enters the interaction through the relation $\kappa ={\left(8\pi \lambda_{B}N_{A}c_{s}\right)}^{\frac{1}{2}}$.

### Titration

Each deacetylated Chitosan monomer represents a weak base, which can be neutral or protonated to a positively charged site. The protonation state of these monomers will depend on the pH of the solution, but also on the local electrostatic environment defined by the charges of the surrounding monomers and *c*_*s*_. A semi-grand canonical ensemble is employed for modeling the titration of the weak base monomers, similar as in previous studies [59–61]. The GlcN CG sites are assigned charge state values *z*_*i*_ = 0 or *z*_*i*_ = 1 according to their protonation state. The free energy contribution from the titration state of the polymer is written as the sum over all *N*_*t*_ titratable monomers in the polysaccharide

$$\begin{array}{c} F^{prot}=\sum_{i\epsilon N_{t}}\mu_{i}z_{i}+\sum_{i\epsilon N_{t}}\sum_{j\neq i}^{N}U_{ij}^{DH}\left(r\right). \end{array}$$ (3)

where the first term accounts for the chemical potential *μ*_*i*_ of protonating the individual monomers and the second term describes the contribution of the screened electrostatic interactions with the other charged sites in the polymer, including also nearest neighbors.

The chemical potential for protonation is related to the pH and the intrinsic dissociation constant of a single site, pK_i_, by $\mu_{i}=k_{\text{B}}T\ln\left(10\right)\left(pH-{\text{pK}}_{\text{i}}^{int}\right)$. As will be discussed below, titration results are sensitive to the value of the intrinsic dissociation constant. The range of experimental values for pK_i_ lies between 6.0 and 7.1. Therefore, here we chose a suitable value for pK_i_ from fitting experimental curves for the degree of dissociation *α* as a function of pH, as described below.

### Simulation protocol

The polysaccharide’s conformational space is sampled with Metropolis MC simulations. Trial conformations were generated using simple Pivot Moves (PM) [62, 63] around the glycosidic bonds. The PM algorithm is simple to implement and a single move results in a large conformational change of the polysaccharide. On the other hand, for collapsed polymers, such large changes can lead to low acceptance rates. However, the present model is only applicable for polymers in solution, so that the advantages outweigh this limitation.

Each PM MC step represents a move of the torsion angles of one randomly chosen glycosidic bond, to a random position on the corresponding PMF map. To maximize sampling of the entire map, no limit on the step size is used. Instead, only steps to regions of the map with energies below a chosen cut-off are attempted, to achieve reasonable acceptance rates. For most cases, this cutoff was chosen to be 7 *k*_B_*T*.

In addition to the conformation altering moves, for each PM MC move *N*_*t*_ protonation moves are performed on randomly chosen titratable sites [60, 61]. This ratio of protonation to pivot moves was found to be sufficient to equilibrate the charge distribution; a further increase of the number of titration moves did not lead to significant changes in the fraction of protonated monomers or the distribution of charges. pH effects on the ionic strength were taken into account. After a successful titration MC moves, the PMF maps for the corresponding links are updated accordingly, as discussed in detail below.

Simulations are stopped and the systems considered to be in equilibrium when the glycosidic angles have sampled all accessible regions of the PMF maps and the physico-chemical properties of the polymer show equilibrium distributions. The number of steps required to achieve this depends on the system conditions, as those determine the acceptance rate.

Simulations were performed with a code based on the cross platform. Net framework Mono [64] and OpenTK libraries [65]. Simulations of a polymer with 1000 monomers including all force field contributions took 24 h for 5000000 PM steps for Chitosan with 90% DD on a Intel(R) Core(TM) i7-3770 CPU 3.40GHz processor.

### Persistence length analysis

The persistence length *L*_P_ is defined by the decay of the bond vector correlation *C*_k_ = 〈**b**_*i*_ ⋅ **b**_**i**+**n**_/|*b*|^2^〉, as *C*_k_ = exp(−*nb*/*L*_P_). For polyelectrolytes, *C*_k_ is determined not only by the stiffness of the bonded structure, but also by the repulsive electrostatic interactions of charged monomers. Therefore, in the context of polyelectrolytes, the concept of an electrostatic persistence length has been introduced [66, 67], to describe the extra chain stiffening due to electrostatic interactions along the backbone, and the contributions to *L*_P_ are often decomposed into an intrinsic part *L*_P,0_, corresponding to the polymer stiffness at infinite *c*_s_, and an electrostatic contribution *L*_P,e_.

**Fig 2:**
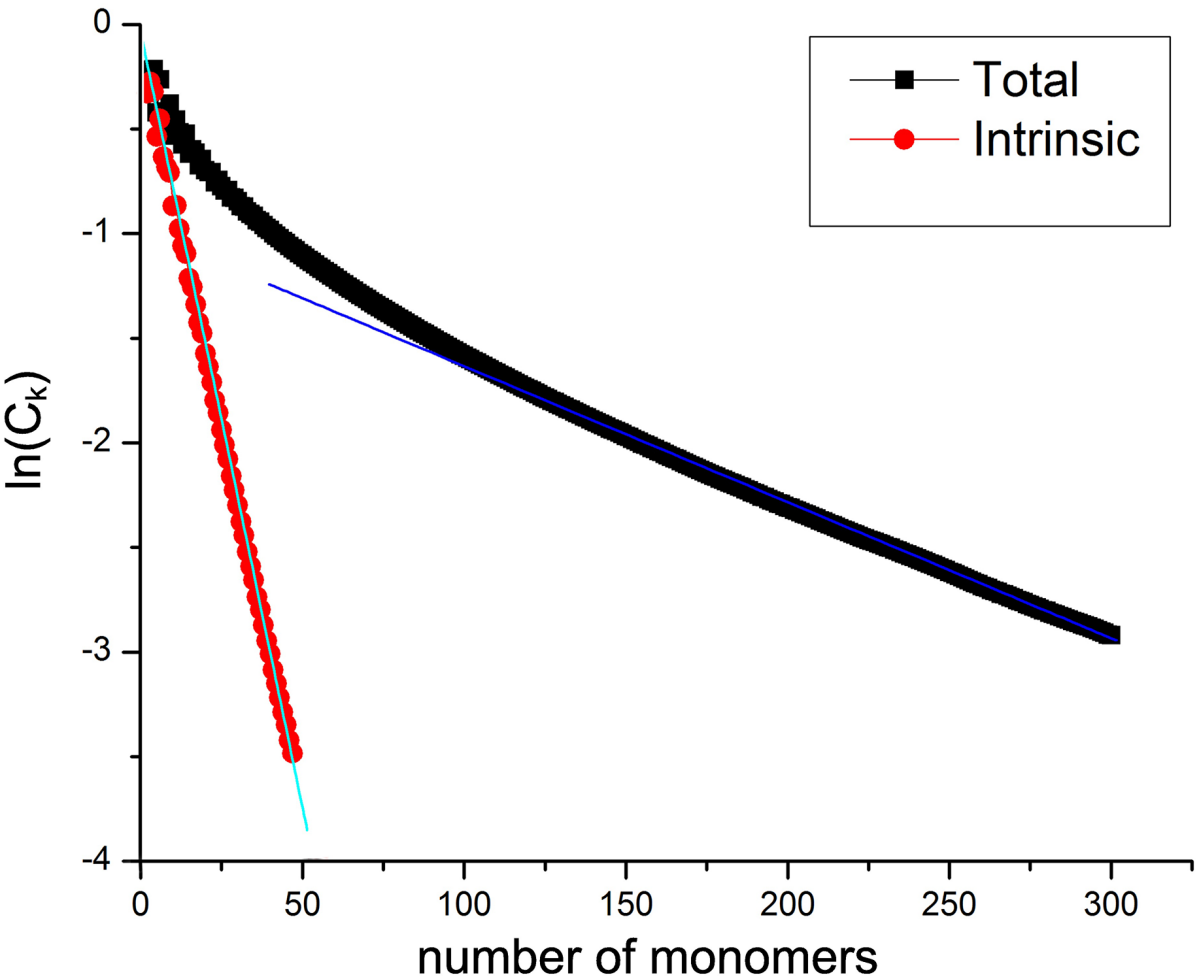
Components of the persistence length. Decay of the bond vector correlation for simulations with and without electrostatic contributions.

As described previously [38], the different contributions lead to different scales in the decay of *C*_k_, as seen in the example shown in Fig 2 for *c*_s_ = 0.001. The different scales make the identification of *L*_P_ from fitting ln(*C*_k_) ambiguous. To determine the influence of the separate contributions to *L*_P_, we obtain *L*_P,0_ from a set of separate simulations without the electrostatic interactions, as illustrated in Fig 2. The values of *L*_P,0_ and *L*_P_ are obtained by fitting ln(*C*_k_) from both simulations. For *L*_P_ the fit is limited to large *n*, where a linear decay is approached.

## Parameter optimization and model validation

### Choice of water model

Although the Amber-Glycam force field has been parametrized in combination with the TIP3P water model, it is frequently also used with the TIP5P water model. Recent MD simulations of saccharide solutions have observed a large effect of the water model on the solution properties [68], and suggested the use of TIP5P water with the GLYCAM06 force field. Based on a comparison of the PMF maps, the internal conformations of the molecules remain relatively unaffected [69]. Here, to evaluate the effect of the water models on the PMF maps as well as the relevance of the resulting differences for the large scale properties of the CG polymer, PMF maps were calculated using the GLYCAM06 force field [42] with a number of different water models. Although all maps show similar features, with the main minima present at the same angles, the conformational space of the maps accessible to thermal fluctuations varies for the different water models. Comparing the radius of gyration data listed in Table 1 shows that such small differences in the dihedral maps can have a measurable effect at the polymer scale. Comparing the results to experimental data [70] suggests that the best agreement can be again achieved using the TIP5P water model, which leads to slightly more flexible polymers. Taken together with previous results, this suggests, that the TIP5P water model is the best available choice, and the maps calculated with TIP5P water are used in the following.

**Table 1:** Radius of gyration from different water models.

Experiment [70]		SPC	SPCE	Tip3p	Tip4p	Tip4pew	Tip5p
DP	*R*_G_(*nm*)	*R*_G_(*nm*)	*R*_G_(*nm*)	*R*_G_(*nm*)	*R*_G_(*nm*)	*R*_G_(*nm*)	*R*_G_(*nm*)
2100	82.5	99.6 ± 25.41	88.1 ± 21.95	97.3 ± 24.7	93.1 ± 23.1	93.1 ± 23.43	77.8 ± 18.27
1500	58.5	81.4 ± 18.78	74.0 ± 16.92	80.8 ± 18.66	79.4 ± 18.72	76.4 ± 17.65	64.6 ± 14.69
1000	47.4	65.7 ± 14.6	60.1 ± 13.18	64.9 ± 14.22	63.8 ± 14.07	61.1 ± 13.51	51.4 ± 11.52
646	34.4	51.1 ± 10.27	47.0 ± 9.86	50.9 ± 10.67	49.0 ± 10.11	48.3 ± 10.122	40.6 ± 8.75
Ave. Diff. (%)		36.8	24.2	35.2	31.4	28.2	10.6

Radius of gyration data for Chitosan polymers of different length using the glycosidic free energy maps obtained with different water models in comparison to experimental data for pH 4.5, *c*_s_ = 0.1. The listed errors correspond to one standard deviation.

### Effect of charge and acetylation on the PMF maps

Chitosan is rarely 100% deacetylated and the deacetylated GlcNH_2_ monomers are weak bases, so that the Chitosan polymers are made up from the three monomeric building blocks GlcNH_2_, ${\text{GlcNH}}_{3}^{+}$ and GlcNac. A previous study at low pH [57] suggested that an acetyl group at the reducing end of a link significantly increases its conformational flexibility, whereas an MD study of infinite Chitosan polymers reports the opposite trend [23].

**Fig 3:**
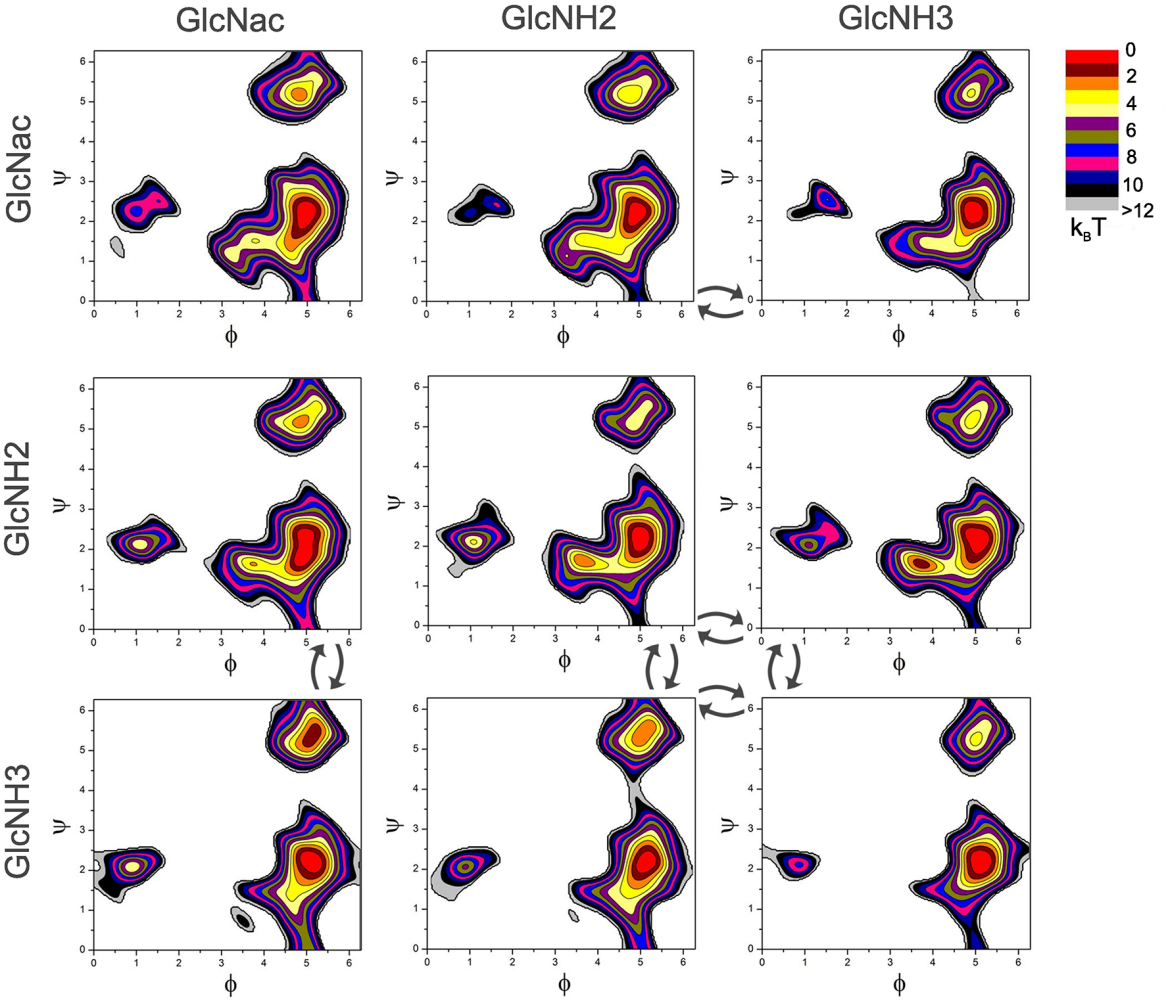
Complete set of nine PMF maps for the nine possible combinations of monomers in a chitosan polymer. Arrows indicate links that can change when the charge on monomer changes in a titration move.

Here we calculated a complete set of nine PMF maps, to include all combinations of charge state and acetylation. The maps, shown in Fig 3 up to 12 *k*_B_*T* all present a similar overall topology, with the main minima present at the same angles. However, both the size of the main minimum and the energy difference to the second minimum, Δ*G*_2_, and thus the population of this second minimum, depends on the link type. Some of the main features contributing to the conformational flexibility of the links are summarized in Table 2.

**Table 2:** Features of the PMF maps for all possible links.

Glycosidic Pair	12kT (%)	<1*k*_B_*T* (%)	*ϕ*	*ψ*	Δ*G*_2_(*k*_B_*T*)
GlcNAc-GlcNAc	29.8 ± 0.93	0.61 ± 0.038	4.956 ±0.015	2.180 ±0.031	2.4±0.144
GlcNAc-GlcNH_2_	27.4 ± 0.72	0.50 ± 0.023	4.933 ±0.017	2.198±0.016	3.0±0.09
${\text{GlcNAc-GlcNH}}_{3}^{+}$	21.7 ± 0.62	0.53 ± 0.012	4.9747±0.0046	2.214±0.0078	3.7±0.076
GlcNH_2_ -GlcNAc	28.0 ± 0.79	0.87 ± 0.036	4.953±0.0047	2.05±0.02	2.6±0.115
GlcNH_2_ -GlcNH_2_	28.4 ± 1.61	0.55 ± 0.0096	4.997 ±0.0034	2.188±0.007	4.0± 0.062
GlcNH_2_ -${\text{GlcNH}}_{3}^{+}$	26.6 ± 0.53	0.68 ± 0.032	5.037 ±0.0148	2.209±0.0278	3.4±0.0914
${\text{GlcNH}}_{3}^{+}$ -GlcNAc	25.6 ± 0.98	0.54 ± 0.011	5.11±0.0176	2.216±0.0448	1.3±0.214
${\text{GlcNH}}_{3}^{+}$ -GlcNH_2_	25.2 ± 1.15	0.49 ± 0.027	5.1287 ±0.0154	2.1736±0.0169	2.2±0.2085
${\text{GlcNH}}_{3}^{+}$ -${\text{GlcNH}}_{3}^{+}$	19.3 ± 0.99	0.60 ± 0.017	5.12 ±0.0154	2.186 ±0.0128	3.6 ±0.155

Accessible area up to 12 *k*_B_*T* and area of the main minimum (<1kT), position of the main minimum and free energy difference Δ*G*_2_ to the second minimum. Error estimates are the STD between PMF maps recorded at different times after 150 ns.

The general features of the maps shown here are in good agreement with the findings obtained in [57]. The maps for the links with a GlcNac monomer at the reducing end, show both the largest main minimum, and the most accessible second minimum, with ${\text{GlcNH}}_{3}^{+}$ -GlcNac showing the lowest energy difference Δ*G*_2_ = 1.35*k*_B_*T*. The link type with the highest Δ*G*_2_ is the neutral GlcNH_2_ -GlcNH_2_ dimer.

Besides the acetylation, the charge of the monomers also has a pronounced influence on the flexibility of the link. A charged monomer at either side tends to lower Δ*G*_2_, whereas the strong electrostatic interactions of two adjacent charged units (${\text{GlcNH}}_{3}^{+}$ -${\text{GlcNH}}_{3}^{+}$) reduce the conformational flexibility.

**Fig 4:**
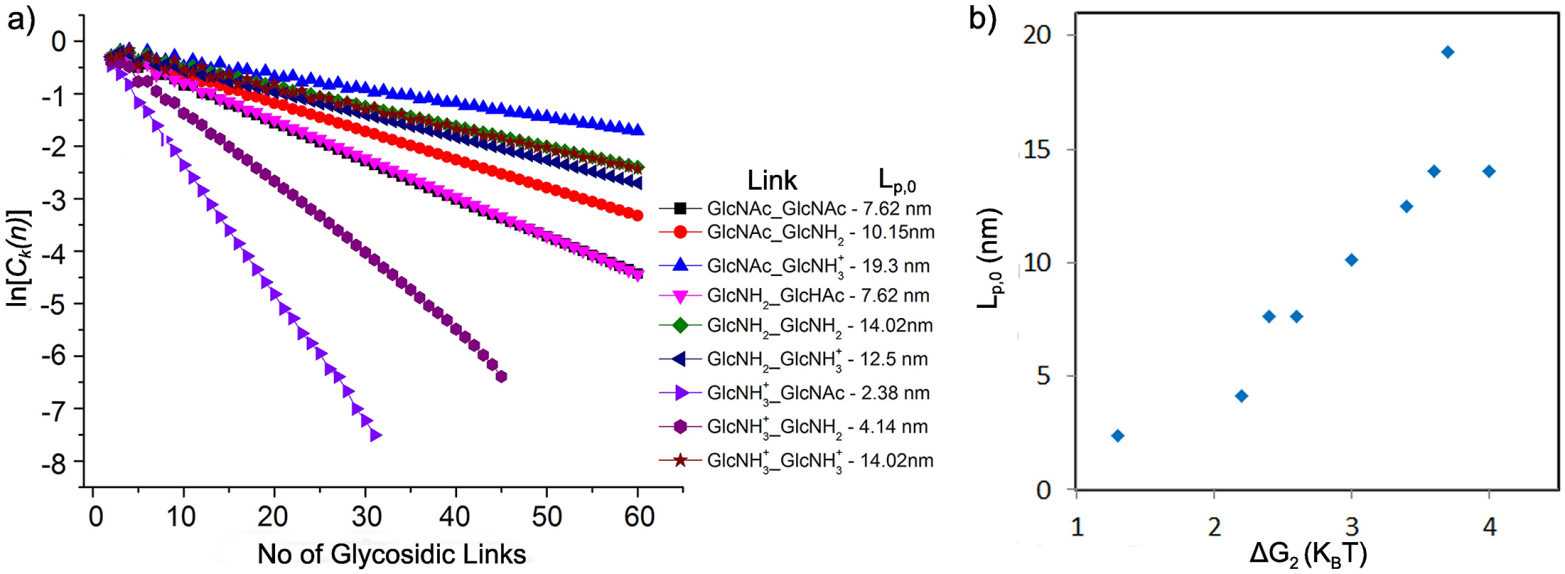
PMF specific persistence length. a) Decay of the bond vector correlation for the nine different link types, and b) the correlation between the polymer stiffness (*L*_P,0_) and the energy of the second minimum (Δ*G*_2_).

To illustrate the link to the flexibility of the polymer, *L*_P,0_ has been calculated for each link type, as shown in Fig 4. The decay of ln(*C*_k_) clearly shows the effect of the map features on the mechanical polymer properties. The data shows that the flexibility is mainly, but not completely, determined by the depth of the second minimum. The most flexible polymers with *L*_P,0_ = 2.38 nm are produced by the ${\text{GlcNH}}_{3}^{+}$ -GlcNAc map, followed by the ${\text{GlcNH}}_{3}^{+}$ -GlcNH_2_ map with *L*_P,0_ = 4.14 nm. These two maps also posses the lowest Δ*G*_2_ (see Table 2).

The highest value of Δ*G*_2_ is found for the GlcNH_2_ -GlcNH_2_ link, and as expected the value *L*_P,0_ = 14.02 nm obtained from this map is much higher. However, other factors, such as the total accessible angular space and other minima are also relevant, as illustrated by the observation, that the stiffness of the links does not in all cases follow the same order as the minimum depth. For instance, the same *L*_P,0_ is found for ${\text{GlcNH}}_{3}^{+}$ -${\text{GlcNH}}_{3}^{+}$ and GlcNH_2_ -GlcNH_2_, while the stiffest polymers, with *L*_P,0_ = 19.3 nm, emerge for the GlcNAc-${\text{GlcNH}}_{3}^{+}$ map.

### Map swapping

Unlike the acetylation state, the charge of the GlcN monomers is not fixed, but can change in the titration moves, to adapt to the solution conditions and the local electrostatic environment of the monomers. The corresponding map changes are indicated in Fig 3 as arrows.

Because the variation in polymer stiffness resulting from the different maps is pronounced, the changes to the link type produced by the titration events may have a significant effect on the polymer properties. To correctly capture this effect, the free energy maps, that can be exchanged should be sampled according to a weighted average of the maps, as PMF_ave = ∑_*i*_*p*_*i*_PMF_i, where *p*_*i*_ are weighing factors, taking into account the probability of each link type in an equilibrated polymer. This probability depends heavily on the degree of dissociation, *α*, but will also be affected by the charges of the neighboring monomers. *α* in turn depends both on the solution conditions and the polymer conformation. Estimates of *p*_*i*_ are therefore difficult to realize. In addition, separate maps would have to be constructed for each setup, making such an approach impracticable.

Instead, a similar effect can be achieved, by updating the type of PMF map after each protonation move. To illustrate the effect of the map swapping procedure, simulation results with and without map swapping are compared to experimental data for Chitosan with low polydispersity [70] in Table 3, using the same solution conditions. At pH 4.5 most monomers will be charged, so that maps for ${\text{GlcNH}}_{3}^{+}$ links were used for the static maps. The comparison shows an improvement by ≈10% for these conditions. Comparing the stiffness of the different links, effects of the map swapping are expected to be the most significant for polymers with roughly 50% of acetylated or deprotonated monomers. The simple updating of the map type works well, because the energy landscapes for all maps are very similar, and the effective averaging has the main effect of broadening of the main minimum. For cases, in which the effect on the free energy landscape are more significant, it may become necessary to also account for the change in the bonded energy of the adjacent links in the protonation free energy Eq (3).

**Table 3:** Effects of map swapping.

Experimental [70]		${\text{GlcNH}}_{3}^{+}$ map		swapped maps	
DP	*R*_G_	*R*_G_	Diff.	*R*_G_	Diff.
2100	82.5	84.4008	2.304	77.9078	-5.67%
1500	58.5	72.4949	23.9229	63.9	9.23%
1000	47.4	57.4462	21.1945	51.37	8.37%
646	34.4	44.8884	30.4895	40.8298	18.69%
Average difference (%)		19.4777		10.49	

Radius of gyration comparison at pH 4.5, *c*_s_ = 0.1 simulated with PMF map swapping (9 PMFs) and without PMF map swapping (using the PMF of ${\text{GlcNH}}_{3}^{+}$ links).

### Acceptance rates and sampling

To obtain sufficient sampling of the conformational space, MC moves should generate a sufficiently large change in conformational space, to obtain fast decorrelation between polymer conformations, while they should at the same time have a sufficiently high acceptance rate. In order to prevent a large number of unsuccessful trial moves while keeping the angular changes large enough to sample other minima, we constrain dihedral angle moves on the PMF maps to regions with energies below a certain cut-off. Cut-off values of 7*k*_B_*T*, 10*k*_B_*T* and 12*k*_B_*T* were tested, and the performance of these maps in terms of both the acceptance rate and polymer properties evaluated.

As expected, the acceptance rate increases for smaller cut-offs. Whereas the fraction of accepted moves for a completely protonated molecule at *c*_s_ = 0.1 using the unrestricted maps is only approximately 1.6%, it increases to 7.5% for the 12*k*_B_*T*, 10% for the 10*k*_B_*T* and 20% for the 7*k*_B_*T* cutoff, respectively. As the probability of sampling parts of the map with Δ*G* ≥ 7*k*_B_*T* is less than 10^−3^, a 7*k*_B_*T* cut-off was chosen. A comparison of the values of *R*_G_ listed in Table 4 shows, that the difference due to the cut-off is minute, compared to the spread of experimental values.

**Table 4:** Radius of gyration from simulation and experiment.

Experimental Conditions				Radius of Gyration (nm)				
ref.	DP	DD (%)	cs	exp.	7kT	full	STD	Diff.
[81]	1223	95%	0.08	45	57.2	57.2	13.2	27.2%
[70]	2100	91%	0.1	82.5	77.9	75.9	17.7	-5.6%
[70]	1500	91%	0.1	58.5	63.9	62.9	14.1	9.2%
[70]	1000	91%	0.1	47.4	51.4	50.4	11.3	8.4%
[70]	646	91%	0.1	34.4	40.8	40.2	8.8	18.7%
[82]	305	91%	0.153	26	23.5	23.2	4.8	-9.5%
[82]	244	91%	0.153	25	21.2	20.9	4.1	-15.4%
[82]	711	80%	0.153	41	36.3	35.7	8.0	-11.5%
[82]	1157	70%	0.153	57	45.3	45.0	10.0	-20.5%
[82]	925	70%	0.153	48	39.1	38.6	8.6	-18.6%
[82]	694	70%	0.153	39	33.8	33.5	7.4	-13.2%

Radius of gyration of Chitosan at pH 4.5 for different ionic strengths, chain length and DD. The listed error estimates for the simulation results are one standard deviation.

**Fig 5:**
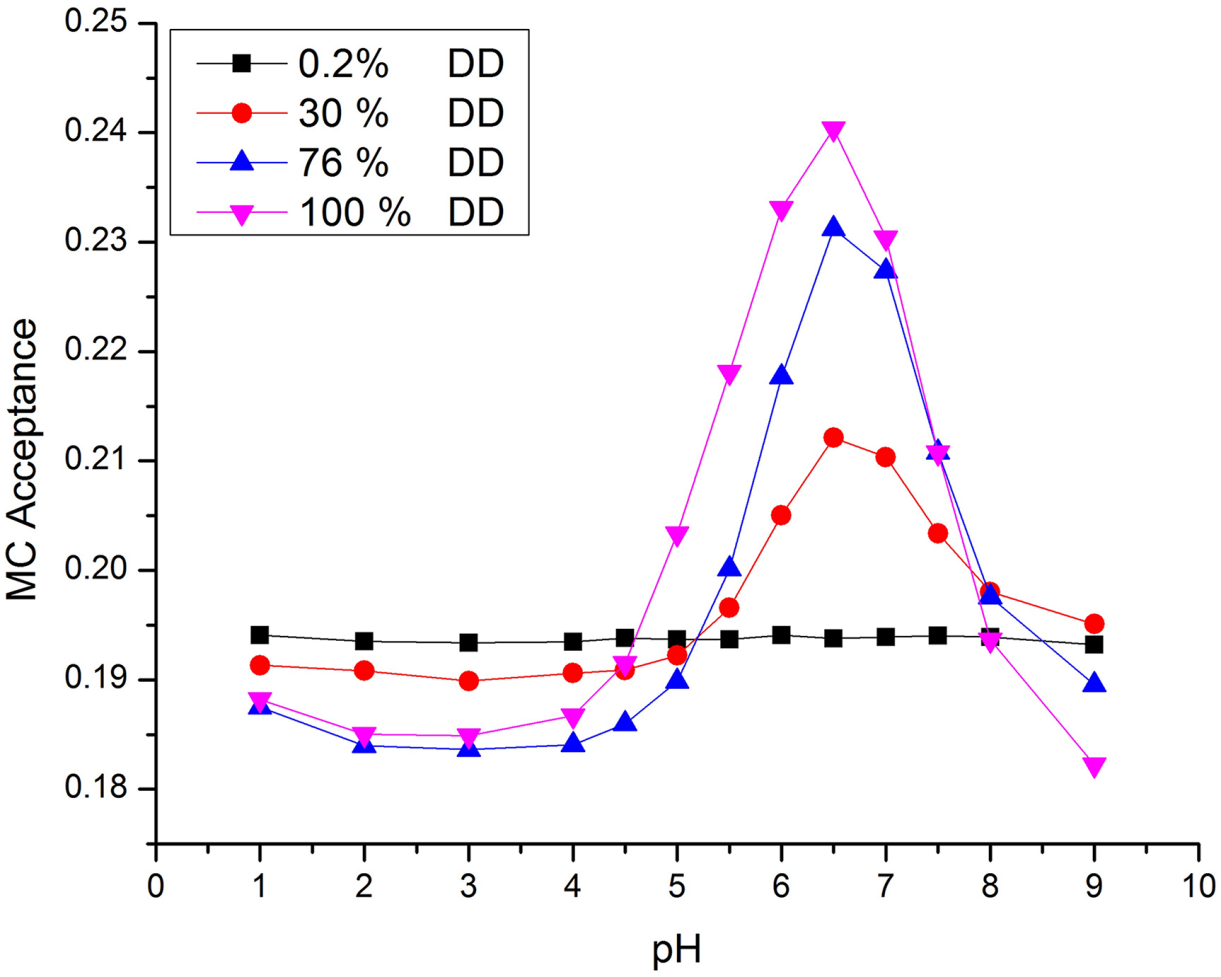
MC acceptance rates as a function of DD and pH.

In addition, the acceptance of the MC moves varies with both the DD and the pH, as summarized in Fig 5 for *c*_s_ = 0.1. At low pH, the strong electrostatic repulsion leads to large excluded volume effects, which reduce the acceptance rate. This effect increases with charge density and thus with DD. Close to the theta point, the transition to neutral Chitosan takes place. In this region, the charge state of a monomer may change frequently, leading to interchange of the maps, and subsequent rearrangement of the links.

### Choice of pK_i_

One of the most important parameter for modeling the titration behavior of the polymers is the intrinsic dissociation constant pK_i_ of the amino group of the Chitosan monomer. This parameter governs the distribution and number of protonated titratable sites.

The value of pK_i_ is often taken to be a characteristic property of the Chitosan polymers. On the other hand, a wide range of experimental estimates for the value of pK_i_ of Chitosan between 6.0 and 7.1 can be found in the literature [71–74]. These values were obtained for different experimental conditions, DD and DP. A systematic study of its variation with DD and DP [75] found, that the DD can affect both the apparent and intrinsic pK values, pK_app_ and pK_i_ of the polymers. pK_i_, is determined by extrapolating the measurements as a function of the degree of dissociation, *α*, to *α* = 1, i.e. to the case of protonating a single site in an otherwise neutral molecule.

In General, the apparent pK value may depend strongly on the monomer’s environment. Important factors influencing this value are the electrostatic environment, dehydration, and hydrogen bond formation [76]. The effect of the electrostatic environment is taken into account in the model by the second term in Eq (3), and in the experimental values through the extrapolation to *α* = 1. The other two factors on the other hand are not accounted for explicitly in the determination of pK_app_ and therefore affects the value of pK_i_.

Both are likely to be relevant for Chitosan, which changes from water soluble at low pH to water insoluble at high pH, and can form many hydrogen bonds, accounting for the variation in the reported pK_i_ values. A complete description of the system may therefore require the implementation of this relation between pK_i_ and the solubility. On the other hand, the simple repulsive interactions limit the applicability of the present model to polymers in solution, for which dehydration and h-bond formation can be expected to play a minor role. Therefore here we have adopted the approach of fitting pK_i_ to best reproduce experimental titration curves and its value was kept constant for all simulations.

**Fig 6:**
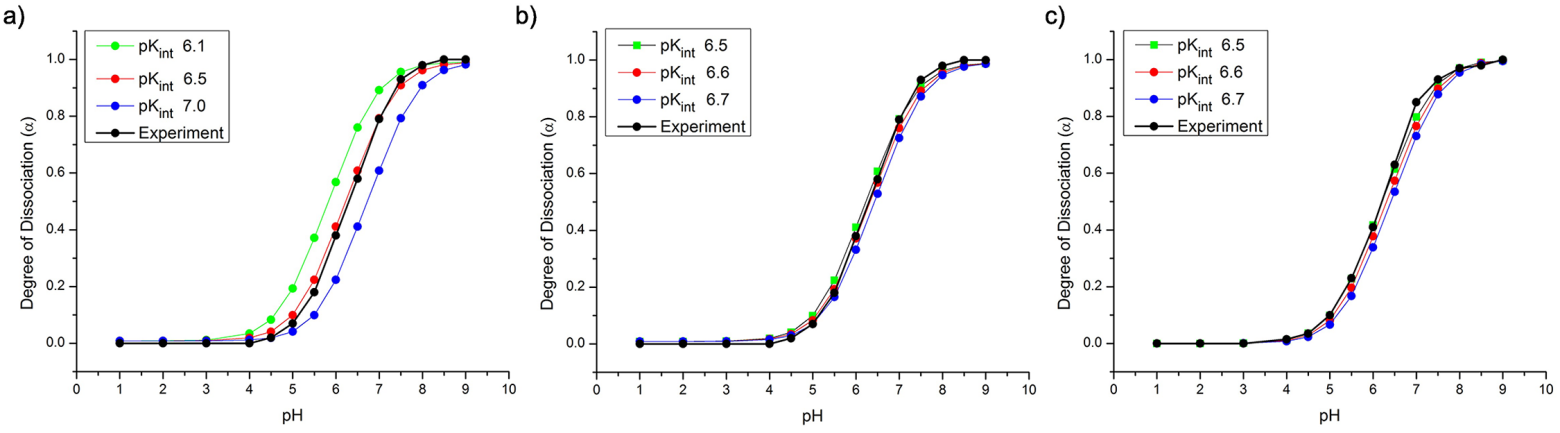
Fitting pK_i_ to titration data. Comparison of simulated and experimental titration data for a) DD = 80% and pK_i_ from 6.1 to 7.0; b) DD = 80% and pK_i_ 6.5-6.7; c) DD = 76% and pK_i_ 6.5-6.7.

For the fit, experimental data for Chitosan with DD 79.8-84.6% [77] was used. Simulations were performed for chitosan polymers with 500 monomers and DD = 80%. The ionic strength for each pH value was set to correspond to the experimental conditions. Titration curves for pKint values 6.1, 6.5 and 7.0, covering the total range of experimental values, are shown in Fig 6(a) together with the corresponding experimental data. The curves illustrate the strong dependence of the charge transition on pK_i_. The pK_app_ of the different curves varies by 2 pH points. Results for a smaller range of pK_i_ values are shown in Fig 6(b), where the the closest agreement is reached for pK_i_ = 6.6. Further comparison with different experimental data set showed, that the pK_i_ value to best reproduce the experimental data varied slightly, between pK_i_ = 6.5 and pK_i_ = 6.7, as for instance in the comparison to 76% DD Chitosan [78] shown in Fig 6(c). A value of pK_i_ = 6.6 produced reasonable agreement in all cases and was chosen for use in the remaining simulations.

### Radius of gyration

After the above fine-tuning of the model parameters, we validate the model against a set of experimental values reported for *R*_G_ at pH 4.5 for different ionic strengths, DD and DP. The results listed in Table 4 shows excellent agreement between simulation results and experiment. The model can reproduce the experimental data within ±20%, with deviations equally distributed in either direction. With the wide spread of experimantal values, due to the polydispersity of the long polysaccharide chains [18, 79, 80], this agreement is as close as can be expected.

## Results and discussion

Now we systematically investigate, how different factors affect the electrostatic and structural properties of Chitosan polymers in solution.

### Effect of *c*_s_ and pH

**Fig 7:**
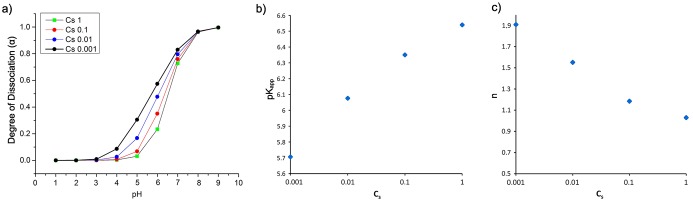
Effect of *c*_s_ on titration. a) degree of dissociation vs pH. b) *pK*_*app*_ and c) *n* parameter as a function of *c*_s_ for a polymer with DP = 1000 and DD = 89.4%.

The ionic strength *c*_s_ of the solution is known to play a significant role in many bio-organic processes, as at high *c*_s_ charges are strongly screened. For Chitosan, the charges on the primary amines that are a key factor governing solubility are screened at high ionic strengths, leading to polymer collapse or aggregation [83]. Especially, in a pH range close to pK_i_, where the charge state of the amines may easily be perturbed by the local environment, screening can have a profound effect on the solubility. The lower the *c*_s_ and thus the screening, the stronger is the coupling between charged monomers along the polymer. This effect can be clearly observed in Fig 7(a) showing the degree of dissociation, *α* as a function of the pH for several values of *c*_s_, where *α* = 0 corresponds to the completely charged and *α* = 1 to the neutral polymer.

With decreasing *c*_s_ the transition from charged to neutral polymers shifts to lower values of pH due to the additional electrostatic energy contribution for protonating the amino-group. Simultaneously, the slope of the transition becomes more gradual, increasing the pH range of partially charged polymers, as compared to the highly screened system. These trends in the transition region are well represented by the modified Henderson-Hasselbach equation [84]

$$\begin{array}{c} {\text{pK}}_{\text{app}}=pH+n*log\left(\frac{1-\alpha }{\alpha }\right) \end{array}$$ (4)

for weak polyelectrolytes. Here, pK_app_ is taken as the pH where half of the titratable monomers are charged.

The prefactor *n* modifies the slope of the curve and accounts for the additional work of charging a monomer against the electrostatic potential from neighboring sites. At high *c*_s_, *n* approaches 1, reflecting the close to complete reduction of the electrostatic repulsion from screening effects. For the lowest *c*_s_, *n* ≈ 2, which significantly reduces the apparent dissociation constant.

**Fig 8:**
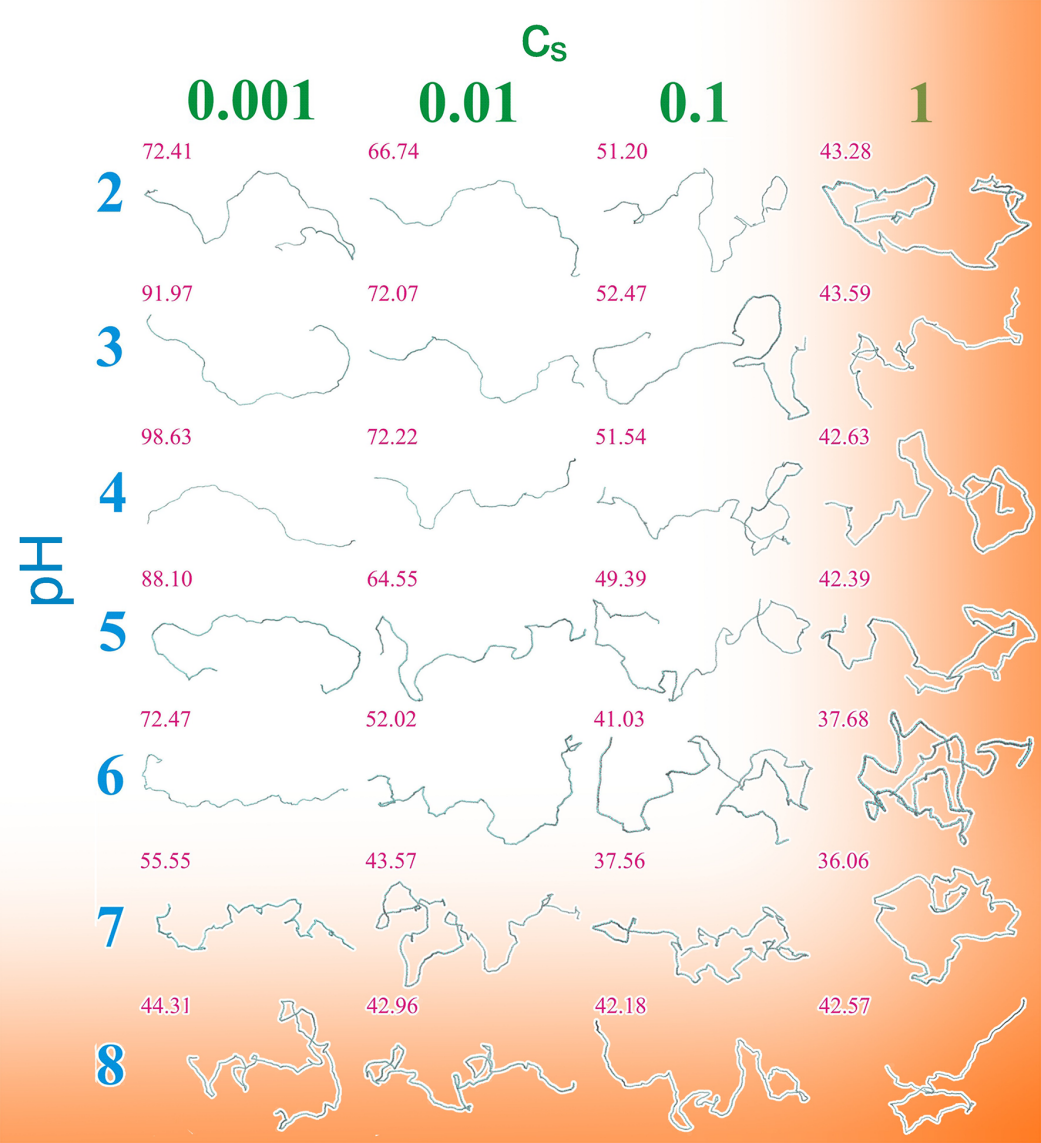
Equilibrium conformations at different pH and *c*_s_ for chitosan with DD 89.4% and DP 1000. Red numbers above each structure gives the root mean square *R*_G_ (nm). Orange regions indicate regions where the conditions for model validity are no longer fulfilled.

As illustrated by Fig 7, the total charge of a polysaccharide is affected by both the pH and the ionic strength of the solution, for a wide range of pH. In addition, the range of interaction of those charges is modulated by *c*_s_. Thus both factors have an impact on the mechanical properties and equilibrium conformations of the polymers. Fig 8 shows representative conformations for polymers with 1000 monomers of 89.4% DD Chitosan for a wide range of pH values and ionic strengths. It is immediately apparent, that the theta point and solubility limit are strongly condition dependent. Whereas, at *c*_s_ = 1 coiled conformations with *R*_G_ close to that at the theta point are observed for the entire range of pH values, at very low *c*_s_ polymers remain extended even at pH 7. Above pH 7 the polymers become completely neutral, and thus insoluble.

Although the CG model presented here lacks detailed attractive interactions between the monomers, which would be required to accurately predict the solubility limits of the polymers, it shows clearly, how the electrostatic interactions governing solubility depend on the solution properties. It is therefore not surprising that the experimentally reported solubility limit for Chitosan differs, with reported values typically in the range of pH = 6 to 6.5 [85], but in some cases Chitosan was found soluble even at pH = 7 [6, 86]. Including attractive interactions or hydrogen bonds in the model may shift the point of collapse to slightly lower pH or slightly lower *c*_s_, however, they are unlikely to play a dominant role under conditions where the polymer is charged.

### Contributions to the persistence length

**Fig 9:**
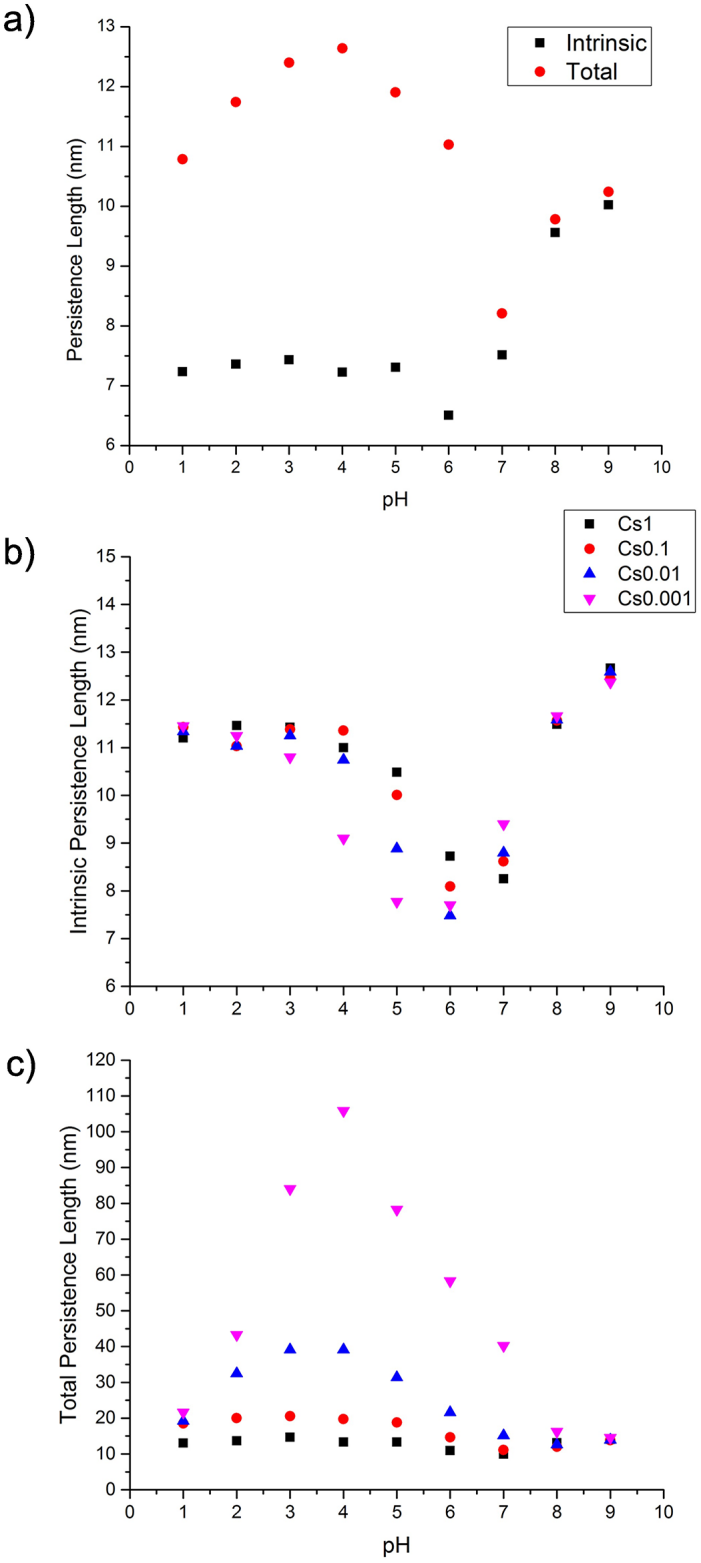
Contributions to the persistence length. a)comparison of *L*_P_ and *L*_P,0_ for *c*_s_ = 0.1 and DD 59%, DP = 500; b) and c) effect of *c*_s_ on *L*_P,0_ (b) and *L*_P_ (c) for 89.4% DD for DP = 1000.

As evident from the snapshots in Fig 8, the orientational correlation of the polymer is strongly affected by the solution conditions. As discussed above, not only the electrostatic contribution *L*_P,e_, but also *L*_P,0_ can be affected by screening effects, since they determine the charge state of the monomers, and thus the flexibility of the links. To gain a better understanding of the factors influencing the persistence length, Fig 9 shows *L*_P,0_ and *L*_P_ as a function of pH for different conditions. The comparison of *L*_P,0_ and *L*_P_ at *c*_s_ = 0.1 shown in Fig 9(a) shows, that the total persistence length for pH values up to pH 6 is significantly larger than *L*_P,0_. The maximum at pH 3-4 arises due to the pH contribution to *c*_s_ at very low pH. *L*_P,0_ remains constant at ≈7 nm for these pH values. The electrostatic contribution decreases dramatically at pH 7 and the total persistence length becomes equal to *L*_P,0_ at pH > 7. In that pH range, *L*_P,0_ first slightly decreases, as the polymer becomes partially charged, increasing the number of flexible ${\text{GlcNH}}_{3}^{+}$ -GlcNH_2_ links, and then increases for neutral polymers at higher pH.

Fig 9(b) and 9(c) show how *L*_P,0_ and *L*_P_ are affected by the ionic strength. The maximum at pH 3-4 increases dramatically for lower values of *c*_s_, reaching values of ≈100 nm for *c*_s_ = 0.001, whereas at *c*_s_ = 1 the electrostatic contribution is negligible even for the highly charged polymer at pH 3 as *κ*^−1^ = 0.3 nm is smaller than the monomer size. In addition, Fig 9(b) shows a clear dependence of *L*_P,0_ on *c*_s_ for pH values between 3 and 7. Comparison to Fig 7 shows that changes in *L*_P,0_ correspond closely to the regime, where the polymer is partially charged. For very low *c*_s_ = 0.001, *L*_P,0_ begins to decrease already at pH 3, and is significantly lower than at higher ionic strengths. This reflects the shift in pK_app_ and *n* observed in Fig 7. The lower charge of the polymer leads to more flexible links and thus smaller *L*_P,0_.

As our model is designed to describe a specific polymer in solution accurately, it presents the opportunity for quantitative comparison with experimental results. In the literature a wide range of values between 5 nm and ≈50 nm [18, 34, 79, 80, 83, 87–94] are reported for the persistence length of Chitosan, which is similar to the range of values observed in Fig 9 for different conditions. As this range of values is rather large, a more detailed comparison is warranted. Especially, although, the above examples show that the persistence length may be strongly condition dependent, most experimental studies were performed using similar conditions with *c*_s_ between 0.1 and 0.2 and pH 4.5 [18, 34, 79, 80, 87, 89, 90, 93, 94], so that other factors must also contribute to the differences.

Experimentally, *L*_P_ is most often estimated from measurements of *R*_G_, by applying the worm like chain(WLC) model. A comparison of several sets of light scattering data obtained with similar conditions showed, that most values of *R*_G_ follow the same dependence on the DP [80, 89, 90]. Other measurements obtained at higher *c*_s_ close to 0.3 follow a similar exponent, but as expected, lower values of *R*_G_ [34, 88, 95]. Thus, the differences in the reported values are more likely due to different approximations used to calculate *L*_P_. Similarly, as our model reproduces the reported values of *R*_G_ well, good agreement for *L*_P_ could be expected. However, here the relevant question is, how the value obtained in analogy to experiment from the WLC model agrees with the microscopic value found from the decay of *C*_k_.

An estimate *L*_P,0_ from the radius of gyration of the polymer in solution, i.e. in a perturbed state, can be obtained following the model of Odijk and Houwart [96], which predicts the electrostatic contributions to both the persistence length and the excluded volume. First, *L*_P_ is calculated using an iterative procedure from the Benoit Doty [97] relation

$$\begin{array}{c} \frac{3R_{\text{G}}^{2}}{L}=L_{\text{P}}\left(1-3\left(\frac{L_{\text{P}}}{L}\right)+6{\left(\frac{L_{\text{P}}}{L}\right)}^{2}-6{\left(\frac{L_{\text{P}}}{L}\right)}^{3}\left(1-\exp^{-L/L_{\text{P}}}\right)\right) \end{array}$$ (5)

For long polymers this is often approximated by the first term

$$\begin{array}{c} \frac{3R_{\text{G}}^{2}}{L}=L_{\text{P}} \end{array}$$ (6)

where *L* is the polymer’s contour length. This estimate of *L*_P_ is used to evaluate the electrostatic excluded volume parameter $z_{el}=\sqrt{\frac{27L}{2\pi }}\kappa^{-1}L_{\text{P}}^{-3/2}$ and the expansion factor $\alpha_{el}^{2}=0.541+0.459{\left(1+6.04z_{el}\right)}^{0.46}$. Then, a better estimate of the unperturbed radius of gyration, *R*_G,0_, is obtained from *R*_G_ = *α*_*el*_*R*_G,0_. This process is iterated until the results converge.

The expression proposed for *L*_P,e_ is

$$\begin{array}{c} L_{\text{P,e}}=\frac{1}{4\lambda_{B}\kappa^{2}} \end{array}$$ (7)

which leads also to an estimate of *L*_P,0_ = *L*_P_−*L*_P,e_. However, the scaling of *L*_P,e_ with *κ*^−2^ is somewhat controversial in the literature. It is predicted by several stiff polymer models [66, 67], whereas other studies predict *L*_P_ ∝ *κ*^−1^ instead [98]. Detailed simulations of discrete chains have varied a large number of parameters to test this relation, and found that, for all cases a quadratic dependence on *κ*^−1^ was observed at large *κ*^−1^ [99]. However, both the proportionality factor and the approach to this relation was found to depend on the polymer model. For the model used here, we find that the microscopic *L*_P_ depends linearly on *κ*^−1^ for all values of *κ*^−1^ used, in agreement with experimental data from [92]. This different behavior is likely to affect the value of *L*_P,0_ predicted from the Odijk model. In addition, In some cases, a polydispersity correction to the z-averaged Radius of Gyration, can lower the estimated value of the persistence length significantly [18, 89]. However, in many studies this is not considered.

Comparing the WLC values to the microscopic *L*_P_ for different *c*_s_ at pH = 4, we find that i) approximating Eq (5) by Eq (6) only works reasonably well at high ionic strength *c*_s_ ≥ 0.1, for polymers with DP ≈ 1000; ii) the values for the total persistence length using the approximation Eq (6) are significantly lower than those found from the microscopic definition. However, the value obtained by the microscopic definition agrees with the one obtained using the full Benoit-Doty relation Eq (5), without applying the excluded volume correction; iii) the values for *L*_P,0_ obtained from the WLC model and Eq (7) agrees well with the microscopic definition for high *c*_s_, where *L*_P,e_ becomes negligible, and for very low *c*_s_, where presumably the proposed *κ*^−2^ relation is approached. For intermediate values however, Eq (7) predicts values of *L*_P,e_ which are small compared to the total persistence length, and therefore much larger values of *L*_P,0_. Using the linear relation *L*_P_ = 7.06 ∗ *κ*^−1^ + 8.6 obtained from fitting the data instead, values of *L*_P,0_ between 7.7 and 10.1 are found for all *c*_s_, which agrees well with the values between 8.5 and 11.5 obtained from the microscopic definition. The estimates of *L*_P,0_ between 6 and 12 nm found by several experimental studies performed at pH 4.5 following this procedure [18, 79, 88, 90, 93] is also in good agreement with these values.

### Effect of the degree of deacetylation

Previous work has shown, that solution properties and self-assembly of Chitosan depend strongly on the DD. Furthermore, also the titration behavior and estimates of the pK_i_ were reported to depend on DD [75]. Three regimes have been found in the dependence on DD of many properties ranging from titration [75] to aggregation [90, 100] and polymer nanostructure [20, 80]. For high DD of 70-100%, the molecules behave as polyelectrolytes, whereas at DD < 50%, the molecules behave as hydrophobic polymers, with independent titratable monomers. At intermediate values a mixture of both effects is observed. As effects of polymer charge, conformation and solubility are intricately connected, with the solubility governed by the electrostatic repulsion, and the charge state being influenced by the local environment, here we try to systematically determine the effects separately.

#### Titration

**Fig 10:**
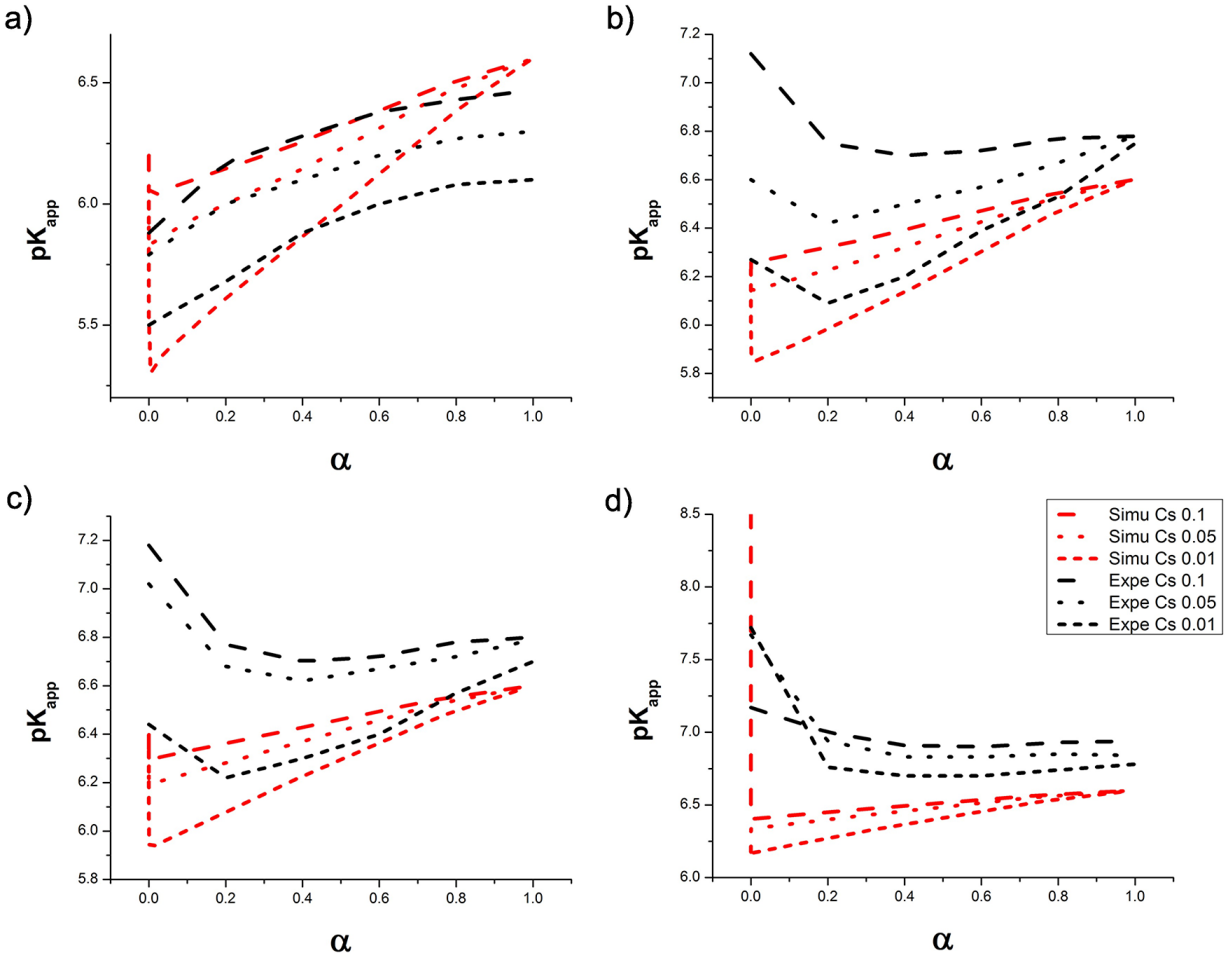
*pK*_*app*_ versus degree of dissociation for different DD of (a) 95%, (b) 68% (c) 50% and (d) 30% for three different values of *c*_s_.

Fig 10 shows a comparison of simulated and experimental titration data, for four different values of DD, ranging from 95% to 30%. Screening effect are apparent at each DD: in all cases pK_app_ decreases more rapidly with *α* at low ionic strength. However, for low values of DD, both the *c*_s_ dependence and the dependence of pK_app_ on *α* become less significant, as the spacing between charges along the backbone is greater, so that screening sets in at much lower *c*_s_.

The data for the highly deacetylated polymers (Fig 10(a) is in good quantitative agreement with the experimental data over a relatively wide range of protonation *α* < 0.6, for all ionic strengths. For higher values of *α*, the simulated curves continue to increase linearly and meet at pK_app_ = pK_i_ for the neutral polymers at *α* = 1. The experimental values for pK_app_ on the other hand begin to deviate from the linear dependence, towards lower values, and different extrapolations for pK_i_ are found for different ionic strengths.

This deviation is related to the limited solubility range of Chitosan. With increasing *α*, Chitosan becomes insoluble and the protonation free energy of the monomers will be affected by dehydration and the formation of hydrogen bonds in addition to the electrostatic environment. In addition, experimental measurement of pK_app_ in the region close to *α* = 0 and *α* = 1, is challenging due to self dissociation of the amino groups close to *α* = 0 and to instability of the pH close to complete neutralization and is based on extrapolation, which may add to the deviation at those values. As discussed above, agreement of the pK_app_ with experimental data can only be expected for soluble polymers.

**Fig 11:**
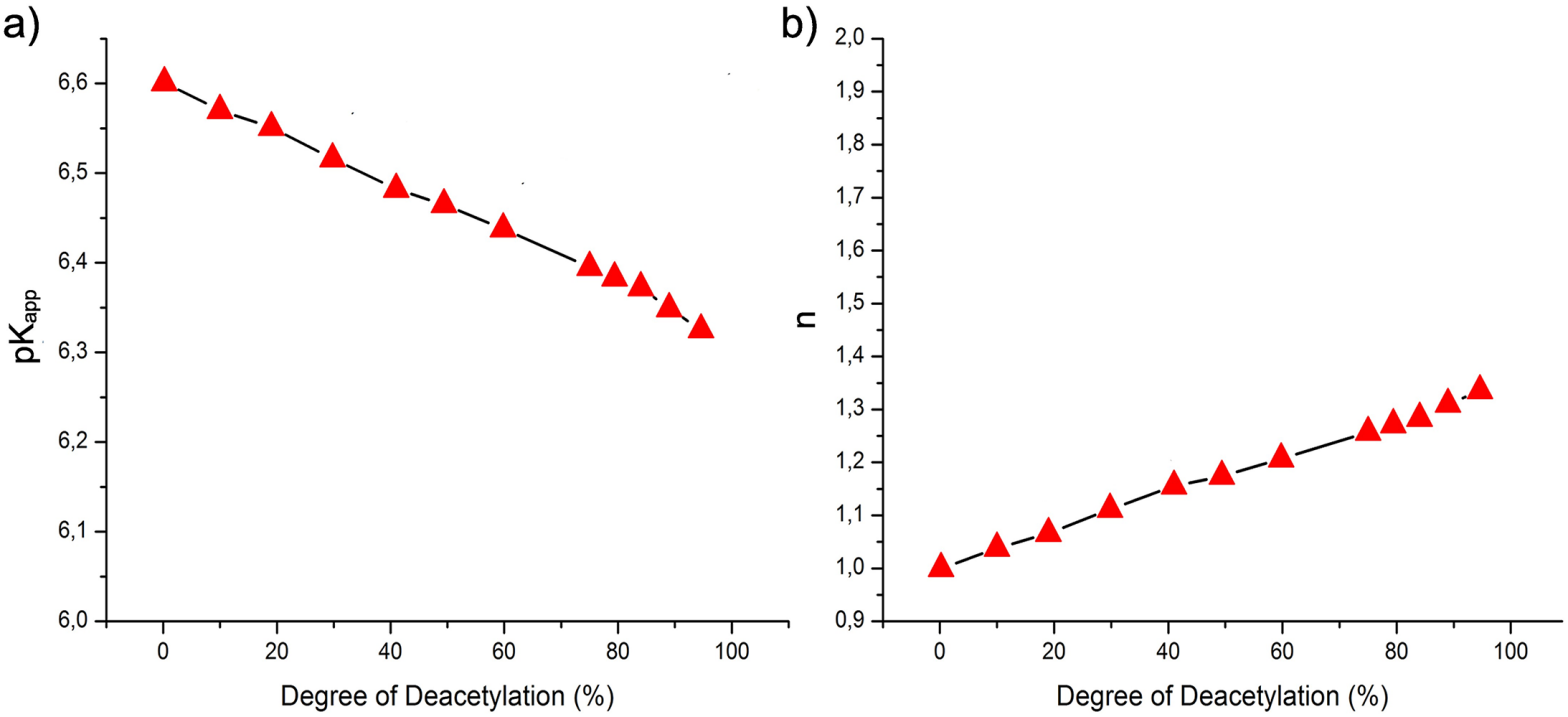
Titration parameters as a function of DD. (a)pK_app_ and (b) *n* parameter from a linear fit of pH vs $\log(\frac{\alpha }{1-alpha})$ in the range $-0.5<\log(\frac{\alpha }{1-alpha})<$ 0.5.

Accordingly, also for polymers with lower DD, the quantitative agreement with experimental data becomes progressively worse (Fig 10(b)–10(d)), as the total polymer charge and thus the solubility range decrease with decreasing DD. None the less, the simulation data captures the qualitative trends of the titration curves well. The relation between titration behavior and DD is summarized in Fig 11, showing pK_app_ and *n* obtained from Eq (4). The curves show a linear dependence of the titration parameters on the DD. Whereas for low values of DD, the titration corresponds to that of independent titratable sites, with *n* = 1 and pK_app_ = pK_i_, at high DD the molecules behave as polyelectrolytes: at *c*_s_ = 0.1 *n* increases to 1.3 close to DD = 100% and pK_app_ decreases due to the nearby charges, and becomes dependent on the solution conditions.

Experimentally, three regimes related to the aggregation behavior of the polymers can be distinguished in the titration data [75, 90]. For high DD of 100-76%, the molecules behave as polyelectrolytes, whereas at DD < 50%, monomers behave as independent titratable sites. For intermediate values of DD, the effects of hydtrophobic association and electrostatics compete. For the simulated data, Fig 11, shows no distinct regimes, but rather a smooth transition from polyelectrolyte to individual charges, reflecting the constant value of pK_i_ used.

#### Structural properties

**Fig 12:**
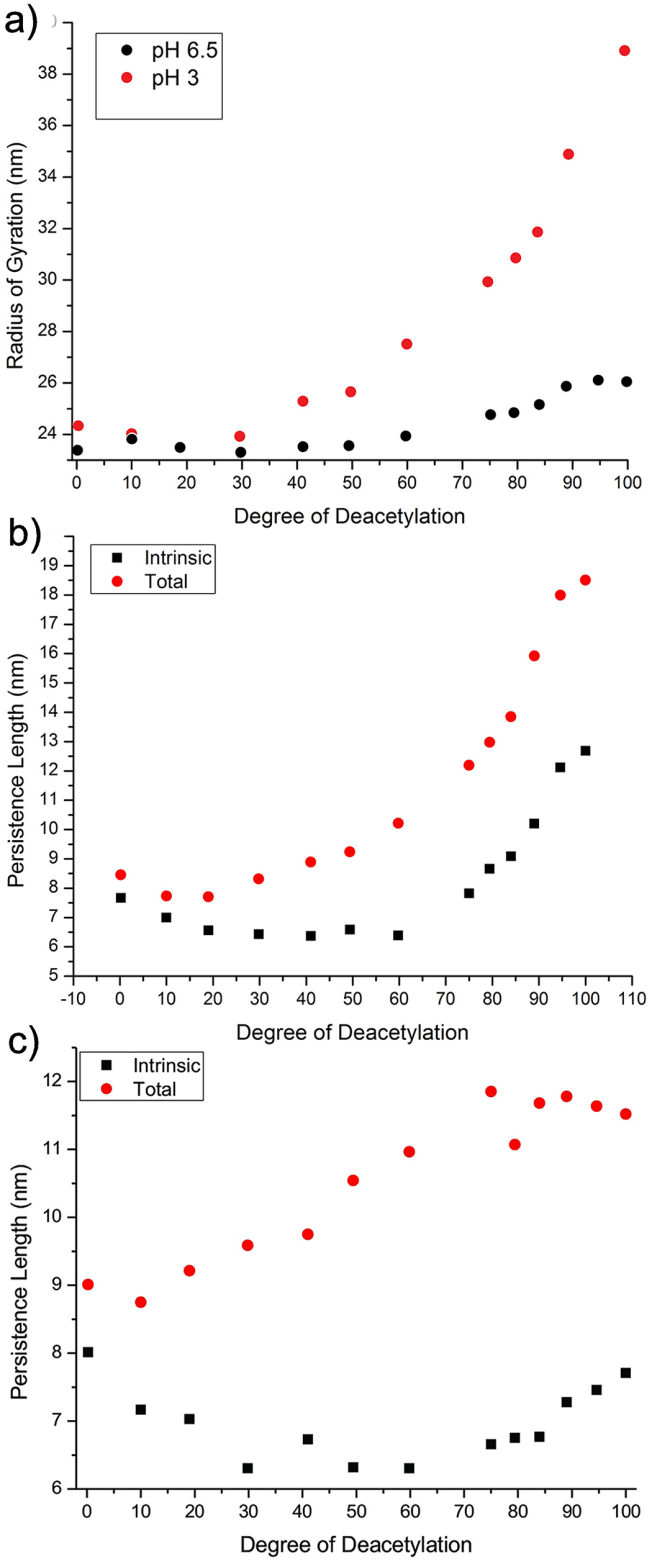
The effect of DD on a) *R*_G_ and (b and c) the contributions to *L*_P_ at pH 4.5 (b) and pH 6.5 (c) for Chitosan with DP = 500.

The topology of the different PMF maps is similar, so that the microscopic structure -local two fold helical structures—is not significantly affected by the DD. Large scale structural properties of Chitosan on the other hand, can depend strongly on the DD. Fig 12 shows for example the radius of gyration as a function of DD for Chitosan at pH = 3 and pH = 6.5 at *c*_s_ = 0.1. For low DD, the two curves look very similar, indicating that up to DD ≈ 40%, the charges along the backbone are spaced far enough apart to be screened to a large degree. For DD ≥ 40%, the polymer at pH = 3 begins to swell, doubling in size for highly deacetylated polymers. Even at pH = 6.5, which corresponds to the minimum in *R*_G_, close to the theta point, some increase in *R*_G_ is observed for DD ≥ 70%, at which point also the increase for pH 3 becomes steeper.

Comparing the average distance between charges along the polymer at the onset of swelling with *κ*^−1^ = 0.96*nm*, this corresponds roughly to two monomer length at *c*_s_ = 0.1 in both cases. At pH 3 all ${\text{GlcNH}}_{3}^{+}$ sites are charged so that at DD = 40%, where the swelling sets in, every second to third monomer is charged. At pH = 6.5 approximately 50% of titratable sites are charged and at DD = 70%, again slightly more than every third monomer is charged. Therefore, in both cases, electrostatic interactions add to the excluded volume starting from approximately the same fraction of charged sites with the average distance between charges slightly larger than *κ*^−1^. This appears reasonable, as both deacetylation and titration are not neccessaryly evenly spaced along the polymer.

An additional contribution to the increase at high DD may come from the intrinsic persistence length, which increases for higher values of DD, as the flexible X-GlcNac links are replaced by stiffer ones. To investigate further, the contributions to the persistence length are shown as a function of DD in Fig 12(b) and 12(c) for pH 4.5 and pH 6.5, respectively. Interestingly, the plots, and especially *L*_P,0_, show three distinct regimes in overall agreement with the regimes observed experimentally for various properties. *L*_P,0_ decreases with DD up to DD = 30%, then remains constant up to 60% DD, after which it begins to increase again. *L*_P,0_ thus has a minimum at intermediate DD, correlated to the high occurance of the most flexible ${\text{GlcNH}}_{3}^{+}$ -GlcNac link. At higher DD, this is progressively replaced by ${\text{GlcNH}}_{3}^{+}$ -${\text{GlcNH}}_{3}^{+}$ at pH 4.5. At pH 6.5, the increase is smaller, as both of the mixed links, ${\text{GlcNH}}_{3}^{+}$ -GlcNH_2_ and GlcNH_2_ -${\text{GlcNH}}_{3}^{+}$ retain more flexibility (see Fig 4). This suggests, that the three regimes observed experimentally in the aggregation behavior, may also be at least partly related to the different flexibility of the glycosidic links.

Experimentally, no different regimes in the polymer stiffness as a function of DD have been reported. There is no general agreement in the literature regarding the dependence of polymer stiffness on the DD. Many systematic studies, find no change in stiffness [88–91]. Others have reported a slight increase [34, 80, 93] with decreasing DD (increasing degree of acetylation). A previous MC simulation study appears to confirm that result [35], whereas our results suggest the opposite trend. This agrees with the decrease in *R*_G_ with the degree of acetylation reported by Lamarque et al. [80] for short chains up to a DP 1600.

In Ref. [35] the maps were obtained using the uncharged GlcNH_2_ monomers only, whereas we have seen, that the presence of a charge can significantly reduce the stiffness of the glycosidic maps (see Fig 4), especially in the presence of GlcNac at the reducing end. One should however also keep in mind, that the increase in *L*_P,0_ with DD observed in this model is produced by the variation in stiffness of the different glycosidic maps. This balance might be rather sensitive to small changes in the free energy landscape, especially for the stiffest or most flexible maps, ${\text{GlcNH}}_{3}^{+}$ -GlcNAC, ${\text{GlcNH}}_{3}^{+}$ -GlcNH_2_ and GlcNac -${\text{GlcNH}}_{3}^{+}$, so that small force field differences may have a large influence on the trends reported here.

### Effect of the pattern of deacetylation

Chitosan is produced by deacetylation of Chitin polymers by treatment in alkali solutions. Properties such as the DD, and the molecular weight depend strongly on the deacetylation process, but are typically well characterized. Much less detail is typically known about the distribution of the acetyl groups, though it is thought that the pattern of acetylation may influence the polymer properties and interactions [101]. Often a random distribution is assumed, however, it has been hypothesized that heterogeneously acetylated chitosan would lead to a blockwise pattern [6, 102, 103]. NMR studies indicate that the acetylation pattern is dominated by a random distribution for a large number of samples, prepared both homogeneously and heterogeneously [101]. However, for highly acetylated samples a tendency towards alternating patterns was found, while for highly deacetylated Chitosan samples, a slight blockwise character was found. Although oligomers with specific patterns of acetylation can be produced by different methods [104–106] these are typically very short and little data with respect to their structural properties is available.

**Fig 13:**
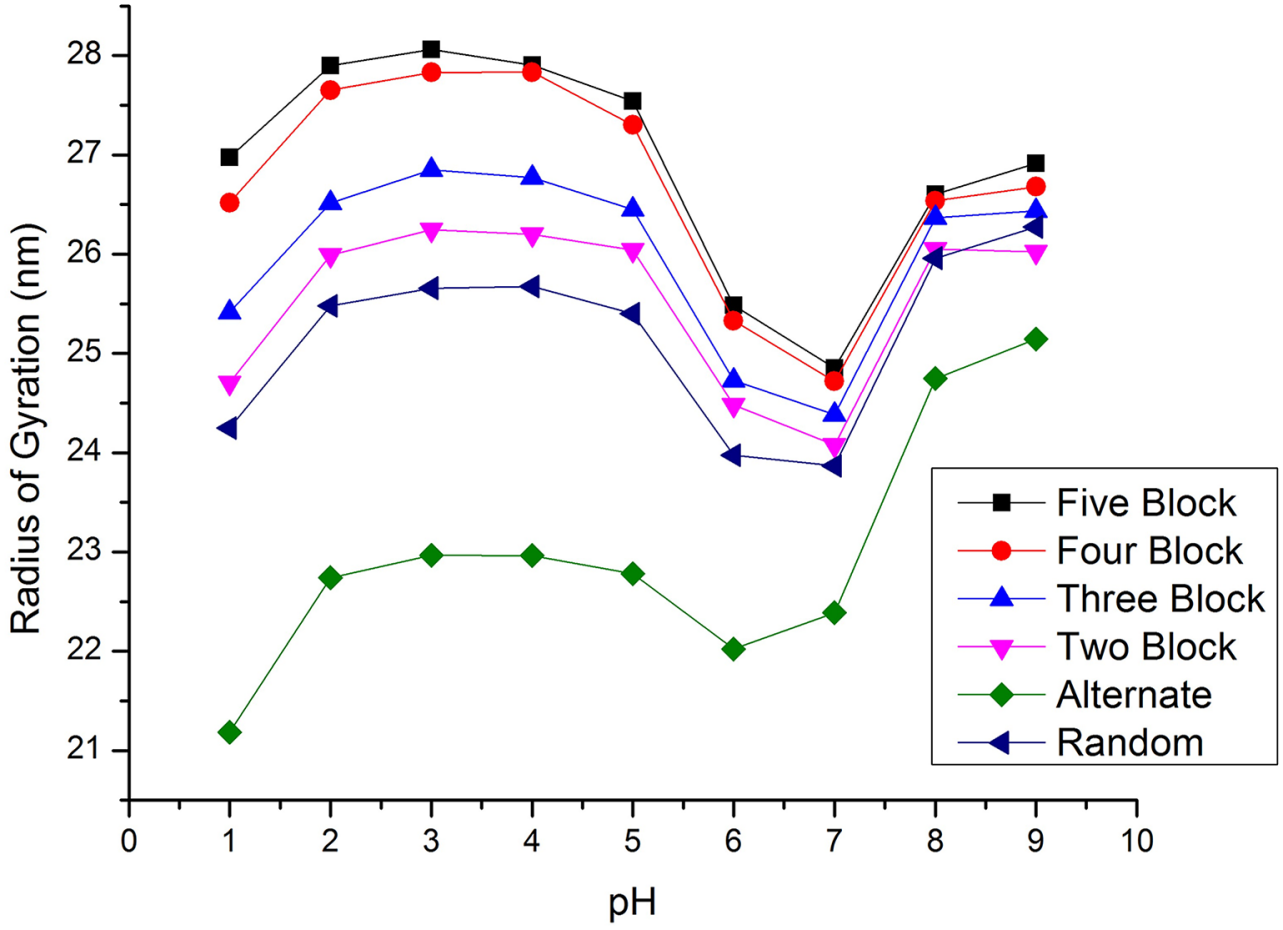
Effect of the pattern of acetylation *R*_G_ of chitosan with 50% DD and DP = 500 for acetylation patterns with different blocksize and a random distribution.

In a computational model on the other hand, different ideal patterns can be constructed easily. Here we have simulated polymers of 50% DD with different acetylation patterns, including alternating as well as blocks of two, three, four and five consecutive monomers, at *c*_s_ = 0.1. The results for the *R*_G_ are shown in comparison to a random distribution in Fig 13. Clearly, the pattern of deacetylation can have a significant effect on the dimensions of the polymers. Especially polymers with an alternating acetylation pattern are significantly more compact than either larger blocks or a random distribution. The swelling of these polymers with respect to the theta point conformations at pH = 6-7 is significantly smaller than for the other acetylation patterns.

For larger blocks, the polymers become more extended, until block sizes of about five monomers, after which polymer properties become more or less block size independent. Again, the opposite trend was predicted by Ref. [35], consistent with the stiffer maps for the neutral GlcNH_2_ -GlcNac monomers, that were used. *R*_G_ for a random distribution of monomers lies only slightly below that of two monomer blocks. One can speculate, that this greater flexibility of polymers with alternating ${\text{GlcNH}}_{3}^{+}$ and GlcNac monomers may be related to the observation that only Chitosan with DD close to 50% could be made water soluble at neutral pH [86, 108].

The more compact conformations for small block sizes can be understood in terms of the distribution of glycosidic links along the polymer. As the intrinsic persistence length for the most flexible glycosidic link ${\text{GlcNH}}_{3}^{+}$ -GlcNAc map is only *L*_P,0_ ≈ 2 nm, whereas for the representative link between deacetylated sites at low pH, ${\text{GlcNH}}_{3}^{+}$ -${\text{GlcNH}}_{3}^{+}$, *L*_P,0_ ≈ 14 nm. In a polymer with alternating pattern, every second link is of the most flexible type, whereas for larger blocks fewer of the very flexible links exist. As a consequence, *L*_P,0_ for an alternating pattern is as low as 3.6 nm but increases to *L*_P,0_ ≈ 6.9 nm for a pattern with block size five. This greater intrinsic flexibility remains apparent, despite the expansion due to electrostatic repulsion. Overall, the expansion due to electrostatic interactions is relatively small, as at this ionic strength *κ*^−1^ = 0.96 nm is only close to the size of two monomers. In addition, the electrostatic expansion is smallest for the alternating pattern, since in that case, all charges are equally spaced and separated by ≈*κ*^−1^, whereas for all other patterns several charges are grouped closer together.

### Chain-Length dependence

**Fig 14:**
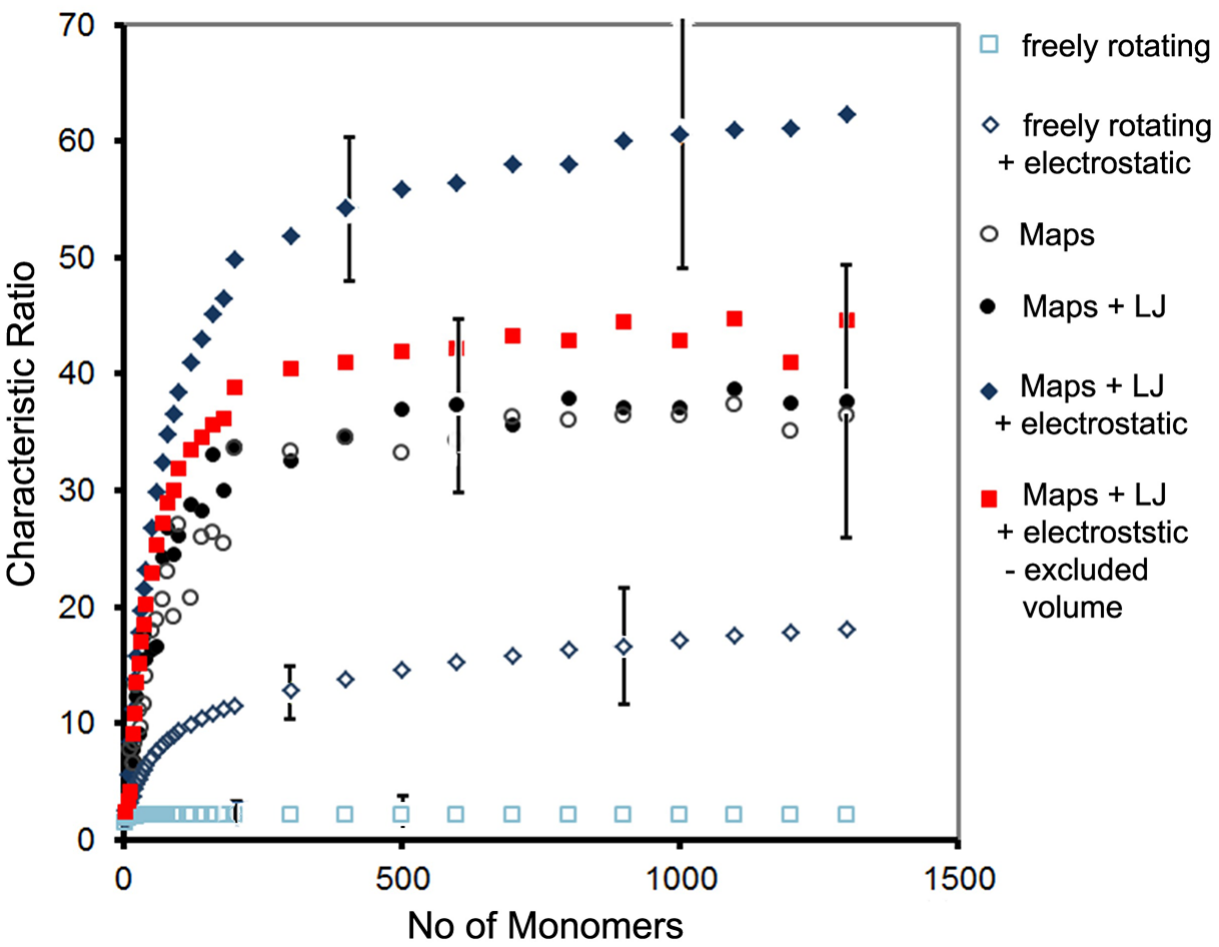
Characteristic ratio vs DP for different force field contributions at pH 4.5 and *c*_s_ = 0.1.

Finally we take a look at the effect of the DP on the polymer properties. To evaluate the effect on the different force field contributions, polymers with DP between four and 1300 monomers were simulated for Chitosan with 95% DD at pH 4.5 and *c*_s_ = 0.1. Fig 14 shows the characteristic ratio *C*_*n*_ = <*R*_ee_>/*nl*^2^ for simulations including different force field contributions, where *R*_ee_ is the end-to-end distance and *l* is the bond length corresponding to one monomer. By definition, *C*_*n*_ usually compares the unperturbed end-to-end distance of the polymer in the theta state to that of a freely jointed chain, *nl*^2^ and thus is a measure of the effect of the chemical bond structure on the polymer properties. Here, we extend its application to compare the effect of different force field contributions, including also electrostatic interactions. Error bars showing 1 standard deviation are included for some points.

As Fig 14 shows, in the absence of electrostatic interactions, *C*_*n*_ reaches a plateau value of 35 nm after *n* ≈ 200 monomers. This relatively large value compared to 2.1 nm for a freely rotating chain with the same bond architecture, illustrates the strong effect of the restricted conformational space of the glycosidic bonds. The addition of steric excluded volume to the maps on the other hand, has a negligible effect. If electrostatic interactions are included, *C*_*n*_ remains polymer length dependent over the whole range, due to the long range of the interactions. Applying the electrostatic excluded volume parameter [96], $z_{el}=sqrt\left(\frac{27L}{2\pi }\right)\kappa^{-1}L_{\text{P}}^{-3/2}$ and using the measured values of *L*_P_ to calculate the expansion factor $\alpha_{R}^{2}=1+1.33z_{el}-2.075z_{el}^{2}+6.459z_{el}^{3}$ derived from perturbation theory [109], these long range effects of the electrostatic excluded volume can be eliminated from the characteristic ratio, and the polymer length dependence disappears. Instead, an only slightly larger plateau than obtained using the maps only is reached. Thus the additional expansion due to electrostatics is well described by excluded volume theory, provided the dependence of *L*_P_ on N is known.

## Conclusion

We present an accurate CG model for equilibrium properties of Chitosan polyelectrolytes in solution, based on the free energy landscapes of the glycosidic bonds and titration of GlcNH_2_ monomers. We show that small variations in the free energy maps for the glycosidic bonds can have a large effect on polymer flexibility, leading to a large range of intrinsic persistence lengths, for the different monomer combinations. As a consequence, in the titration moves, we take into account the changing protonation state of the monomers, by updating the corresponding free energy map.

This makes the response of the model to solution properties more precise, and has a strong influence on both structural and electro-chemical properties of the molecules, especially under conditions, where monomer charges are likely to change. The model is shown to accurately reproduce available experimental data for conditions, for which Chitosan is soluble. For Chitosan aggregates, both changes to pK_i_ and missing specific interaction potentials reduce the quantitative accuracy of the results.

We apply the model to systematically investigate the effect of different solution properties and force field contributions to the polymer properties. In particular, we also investigate the effect of degree and pattern of acetylation, for which widely different experimental trends are reported. Both can significantly affect the polymer properties, depending on the solution conditions. For structural properties, three regimes are found for the dependence on DD, similar to those found experimentally for a variety of different properties, related to solubility. This suggests, that the differences in flexibility of links involving GlcNac may be part of the origin of this observation.

## Supporting information

S1 File Glycosidic free energy maps. Numeric data for the free energy maps of all glycosidic link types, and CG input structure with 2200 Monomers.
(ZIP)
